# The Diversification of the LIM Superclass at the Base of the Metazoa Increased Subcellular Complexity and Promoted Multicellular Specialization

**DOI:** 10.1371/journal.pone.0033261

**Published:** 2012-03-15

**Authors:** Bernard J. Koch, Joseph F. Ryan, Andreas D. Baxevanis

**Affiliations:** Genome Technology Branch, National Human Genome Research Institute, National Institutes of Health, Bethesda, Maryland, United States of America; Université Paris-Sud, France

## Abstract

**Background:**

Throughout evolution, the LIM domain has been deployed in many different domain configurations, which has led to the formation of a large and distinct group of proteins. LIM proteins are involved in relaying stimuli received at the cell surface to the nucleus in order to regulate cell structure, motility, and division. Despite their fundamental roles in cellular processes and human disease, little is known about the evolution of the LIM superclass.

**Results:**

We have identified and characterized all known LIM domain-containing proteins in six metazoans and three non-metazoans. In addition, we performed a phylogenetic analysis on all LIM domains and, in the process, have identified a number of novel non-LIM domains and motifs in each of these proteins. Based on these results, we have formalized a classification system for LIM proteins, provided reasonable timing for class and family origin events; and identified lineage-specific loss events. Our analysis is the first detailed description of the full set of LIM proteins from the non-bilaterian species examined in this study.

**Conclusion:**

Six of the 14 LIM classes originated in the stem lineage of the Metazoa. The expansion of the LIM superclass at the base of the Metazoa undoubtedly contributed to the increase in subcellular complexity required for the transition from a unicellular to multicellular lifestyle and, as such, was a critically important event in the history of animal multicellularity.

## Introduction

LIM is an ancient eukaryotic protein domain that originated prior to the last common ancestor of plants, fungi, amoebae, and animals. The domain name is an acronym of the first three genes in which it was identified: Lin-11 from *Caenorhabditis elegans* [1], Isl1 from rat [2], and Mec-3 from *Caenorhabditis elegans* [3]. LIM domain-containing proteins participate in cytoskeletal complexes such as focal adhesions and adherens junctions to regulate cell growth, motility, and division (reviewed in [4], [5], [6]). Many LIM proteins also shuttle to the nucleus, where they regulate gene expression and cell fate decisions [6], [7]. Given their roles in focal adhesion dynamics, LIM proteins are prominent in tissues having elevated levels of cell-cell interactions (e.g., striated muscle; reviewed in [8], [9]). In addition, their influence on intercellular communication makes them crucial to processes involving complex cellular navigation (e.g., axon guidance; [10]). It is, therefore, unsurprising that LIM proteins are implicated in a variety of heart and muscle conditions, neurological disorders, cancers, and other diseases [11], [12], [13], [14], [15], [16].

The LIM domain is 50–65 amino acids in length and is defined by two cysteine-histidine-rich zinc fingers separated by a hydrophobic linker. The defining feature of the domain is its eight structural zinc-coordinating residues (usually cysteines). Outside of these highly conserved residues, LIM domains are highly diverse and lack a consensus protein-binding sequence (reviewed in [6]). In terms of diversity of domain architectures, LIM domains are considered to be amongst the most promiscuous [17]. In comparison to those found in plants, animal LIM proteins are particularly numerous and diverse in their architectural complexity [18], [19], [20].

In humans, the LIM superclass has been previously divided into established groups based on sequence and characteristic domain architectures. These groups have been further subdivided into at least three categories based on function, domain architecture, and cellular localization [6], [7], [21]. Two of these reviews classified individual LIM domains by sequence similarity. However, promiscuity and low sequence conservation make it difficult to resolve homologous relationships between LIM domains without rigorous phylogenetic analyses. There have been few evolutionary studies aimed at deducing the relationships between LIM groups (e.g., [22]), and only LHX has been extensively characterized outside of the Bilateria [23].

**Figure 1:**
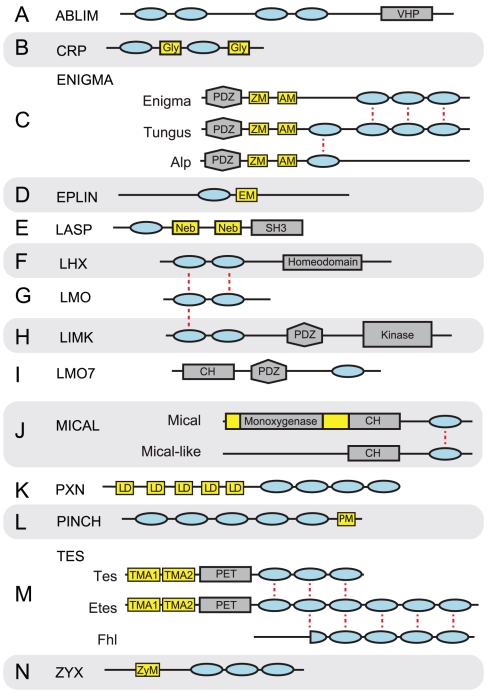
Domain architectures of LIM superclass of proteins. LIM domains are represented as blue ovals, non-LIM PFAM domains as grey shapes, and motifs and conserved regions as yellow boxes. In each case, the order of the domains or motifs is correct, but the spacing and length is not to scale (see Table S1 for actual coordinates). LIM domains from one class or family that appear to be related to another LIM domain from another class or family are connected with a red dashed line. Abbreviations are as follows: villin headpiece domain (VHP), glycine rich region (Gly), zasp motif (ZM), alp motif (AM), EPLIN motif (EM), nebulin repeat (Neb), SRC homology 3 domain (SH3), homeodomain (HD), calponin homology domain (CH), leucine-aspartate repeat (LD), PINCH motif (PM), TES motif A1 (TMA1), TES motif A2 (TMA2), ZYX motif (ZyM). For loss events see Table S1.

In this study, we analyzed 623 LIM domains in 265 proteins from six animals and three animal-related unicellular eukaryotes using a phylogenetic approach. We used phylogenetic groupings of LIM domains, along with domain architectures and motif signatures, to classify 206 of the LIM proteins into 14 LIM classes (Fig. 1). Our evolutionary classification of the LIM superclass shows that there was a major expansion of these proteins in terms of the number of classes and the architectural complexity of the superclass just prior to the last common metazoan ancestor. Given the prominent role that LIM proteins play in connecting nuclear transcription with extracellular signals, the expansion of this superclass was likely a critical step in the establishment of the kind of subcellular complexity required for animal multicellularity.

## Results

### Overview of LIM domain identification and classification

In the course of this study, we adopted the classification scheme previously put forth for homeodomain proteins [24]. In this scheme, a class contains one or more families that, in turn, contains one or more proteins. A protein family is usually defined as containing all proteins that descended from a single ancestral protein in the last common ancestor to bilaterians, while classes reflect deep evolutionary relationships between multi-domain proteins with distinct domain architectures. We divided the previously defined groups of LIM domains into 14 classes (ABLIM, CRP, ENIGMA, EPLIN, LASP, LIMK, LHX, LMO, LMO7, MICAL, PXN, PINCH, TES, ZXN). The term “superclass” is used to refer to the entire repertoire of LIM proteins.

**Figure 2:**
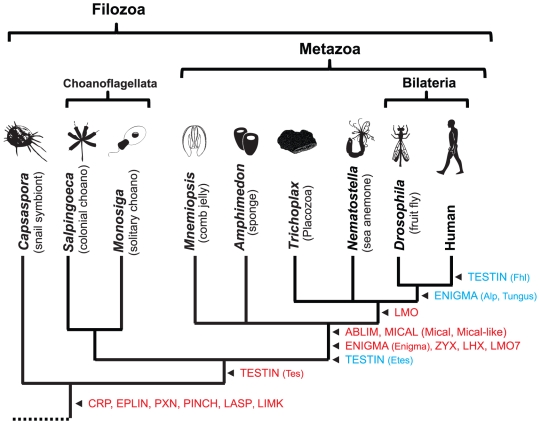
Origin of LIM classes and families. Arrows indicate the stem lineage where a particular group of LIM proteins originated. Classes are denoted in capital letters and are not shown in parentheses. Families are denoted in lower case and appear after the class. The first appearance of a class is in red, while subsequent appearances of families of that class are in blue. The tree is based on the ParaHoxozoa hypothesis [109]. The phyla represented are as follows: *Capsaspora ocwazarki* (Filasterea), *Salpingoeca rosetta* (Choanoflagellatea), *Monosiga brevicollis* (Choanoflagellatea), *Amphimedon queenslandica* (Porifera), *Mnemiopsis leidyi* (Ctenophora), *Nematostella vectensis* (Cnidaria), *Trichoplax adhaerens* (Placozoa), *Drosophila melanogaster* (Arthropoda), and *Homo sapiens* (Vertebrata).

**Figure 3:**
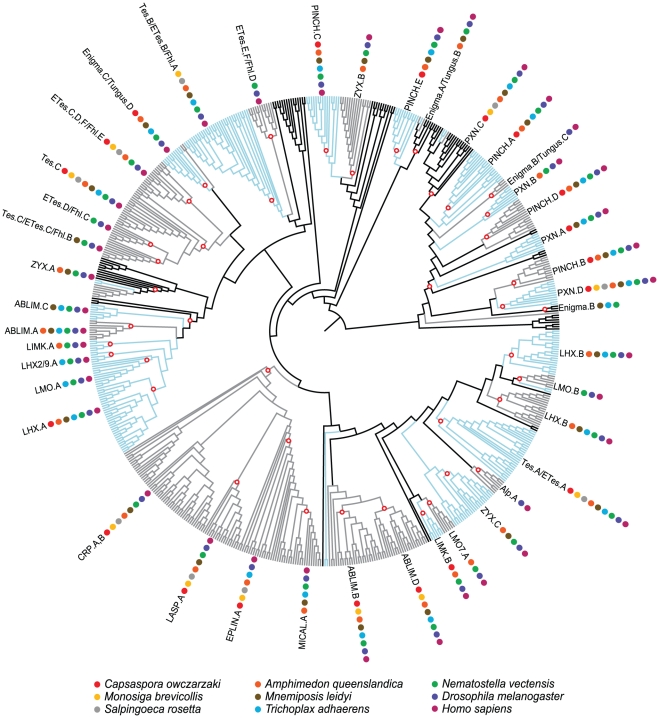
LIM domain cladogram. Alternating blue and grey coloring delineates homology groups; black regions are unclassified. For the homology group of each taxon, see Table S3. White circles with red outlines denote visually identified clades that contain a specific LIM domain conserved within a class or family. Colored circles indicate which species have taxa present within that manually annotated clade. For tip labels, branch lengths, and bootstrap values see Figures S2 and S3.

We used the LIM hidden Markov model (HMM) from PFAM [25] as a query against nine predicted proteomes – *Capsaspora ocwazarki* (Filasterea), *Salpingoeca rosetta* (Choanoflagellatea), *Monosiga brevicollis* (Choanoflagellatea), *Amphimedon queenslandica* (Porifera), *Mnemiopsis leidyi* (Ctenophora), *Nematostella vectensis* (Cnidaria), *Trichoplax adhaerens* (Placozoa), *Drosophila melanogaster* (Arthropoda), and *Homo sapiens* (Vertebrata); see Figure 2 for the relationships between these species. We retrieved a total of 623 LIM domains from 265 proteins and constructed a multiple sequence alignment by aligning each individual sequence to the LIM HMM. We then used this alignment (shown in Fig. S1) and multiple starting trees to generate phylogenetic trees under both Bayesian inference and maximum likelihood frameworks. The maximum likelihood of each of these trees was evaluated, and the tree with the highest likelihood was selected for further analysis (Fig. 3, S2 and S3). This process was also performed on an alignment consisting of only human LIM sequences (Fig. S4 and S5). For both datasets, we generated 100 bootstrap replicates, finding poor support for most clades.

Given this poor statistical support, we used a consensus approach to identify consistently recovered clades. We generated a strict consensus tree between a pruned version of the multi-species tree and the human-only dataset. We designated each of the 38 clades radiating from the midpoint of this strict consensus tree as human LIM homology groups. Out of 171 human LIM sequences, only 12 were placed in homology groups with three or fewer taxa. Superimposing these homology groups onto the multispecies tree in Figure 3, we placed 392 of the 473 non-human LIM sequences into these homology groups using a nearest neighbor approach (see Methods). The 59 proteins that could not be classified shared a most recent common ancestor with human taxa from multiple homology groups and did not belong to a lineage diverging just outside of a single-homology group clade (See the “Unclassified” section of Table S1).

We retrieved the full amino acid sequences of all 265 hypothetical proteins and scanned them for non-LIM PFAM domains using HMMER [25], [26]. We also scanned these sequences for motifs using the motif discovery program MEME [27]. We used the following criteria to define the domain architecture of a particular LIM protein: (1) the number of LIM domains, (2) the presence of any non-LIM PFAM domains, (3) the presence of any sequence motifs, and (4) and the arrangement of these features. We used these domain architectures, along with the assignment of each LIM domain into one of the homology groups described above, as parallel lines of evidence to systematically place each protein into one of the 14 LIM classes (Table S1).

### ABLIM class

ABLIM genes code for focal adhesion and adherens junction scaffolding proteins that mediate interactions between actin filaments and cytoplasmic targets; they also activate cytoskeletal signaling cascades that lead to transcription [28], [29], [30]. These proteins consist of a carboxyl-terminal villin headpiece (VHP) domain and four amino-terminal LIM domains (Fig. 1A). The domain architecture of ABLIM proteins makes them important components for cell-cell adhesion in epithelial tissues; the VHP domain confers F-actin-binding properties, while the LIM domains localize these proteins to adherens junctions [29]. Defects in the *Drosophila* ABLIM protein unc-115 lead to axon navigation errors [31].

In addition to the three human ABLIMs, we found a single ABLIM in *Drosophila*, *Nematostella*, and *Amiphimedon* with the canonical architecture of four LIM domains and a VHP domain (Table S1). *Mnemiopsis* has two ABLIM proteins: one containing a VHP and one without. Similarly, *Trichoplax* has two ABLIM proteins that are both missing the VHP domain. One of the *Trichoplax* ABLIMs is also missing the most carboxyl-terminal LIM. *Capsaspora*, *Monosiga*, and *Salpingocea* do not have ABLIM proteins, suggesting that ABLIM is a metazoan novelty (Fig. 2).

### CRP class

CRP is an ancient class of LIM proteins. It is the only LIM class that includes proteins from plants and the amoeba *Dictyostelium discoideum* [19], [20], [30], [32]. As in plants, animal CRP proteins have been reported to modulate cytoskeletal dynamics [19]. CRP proteins stabilize α-actinin [33] and are involved in scaffolding at focal adhesions [34]. They also can shuttle to the nucleus where they serve as transcriptional regulators [32]. A CRP gene in *Nematostella* is expressed in the developing mesenteries, the coelenteron lining, and tentacles – all muscle-associated tissues [35].

CRP proteins typically contain two LIM domains separated by an approximately 50-residue linker, although some class members contain only a single LIM domain (Fig. 1B). A conserved 15–20 amino acid glycine-rich motif can be found on the carboxyl-terminus of each LIM domain [7]. In human CRP1, this motif is required for its localization to the cytoskeleton and ability to bundle actin [36]. This region may also overlap with a CRP nuclear localization signal [32].

If we root our multi-species tree with CRP, which is reasonable given that CRP is present in plants, the LIM domains of this class form a clade that is almost monophyletic (Fig. 3, S2 and S3). All but four of the proteins within this clade have a glycine-rich motif. Two of these four (Nv_68197, and Aq_223000) appear to be partial isoforms from CRP proteins that are already represented in our dataset (Nv_78916 and Aq_229999). We consider these proteins to be misannotated and have removed them from our table of classified LIM proteins (Table S1). An alternative gene model for the single LIM protein Nv_7949 encodes two CRP LIM domains and a glycine-rich motif. Therefore, we have designated this protein as belonging to the CRP class. We have classified Co_04145T0 (from *Capsaspora*) as “unclassified” rather than a *bona fide* CRP, since we are unable to generate any corroborating evidence to ally this protein with the CRP class.

We identified six CRP proteins in humans, eight in *Nematostella*, one in *Mnemiopsis*, two in *Amphimedon*, and two in *Capspaspora* (Table S1). Two *Drosophila* CRP-related proteins each contain five tandemly duplicated LIMs and glycine-rich motifs. We were unable to unambiguously recognize CRP proteins in *Trichoplax*, *Salpingoeca*, or *Monosiga*.

### ENIGMA class

The ENIGMA class consists of three families with differing numbers of LIM domains; Alp family proteins have one, Enigma family proteins have three, and Tungus family proteins have four (Fig. 1C). The proteins of this class include a PDZ domain that binds α-actinin and modulates actin dynamics. ENIGMA proteins are able to enter the nucleus to modulate gene expression and signal transduction (reviewed in [37], [38]).

In addition to the LIM and PDZ domains, two motifs have been described in a subset of the ENIGMA class of proteins. The Zasp (ZM) motif helps localize the Pdlim7 protein to α-actinin [39]. Using the HMM from the SMART database [40], we identified this motif (Table S2) in the *Drosophila* Tungus protein, the human Alp proteins Pdlim1 and Pdlim3, as well as in the human Enigma protein Ldb3 (Table S1). This suggests that this motif was established prior to the divergence of the Alp, Enigma, and Tungus families.

A second motif of unknown function, the Alp motif (AM), was previously thought to be present only in the Alp family of proteins (e.g., human Pdlim1-4) [22]. However, we find that most of this motif is conserved in all members of the human Enigma family (Pdim5, Ldb3, and Pdlim7). In addition, we recovered the Alp motif in *Nematostella* and *Mnemiopsis* Enigma proteins (Nv_ 231944, Ml_108023b), as well as a Tungus protein encoded by the cephalochordate *Branchiostoma floridae* (Bf_123730). This suggests that this motif was also established prior to the divergence of these three families.

In *Drosophila*, a single ENIGMA class protein, Tungus, exists with a PDZ domain and four LIM domains. The first Tungus LIM forms a clade with the LIM domain from the Alp family, while the other three LIM domains are related to each of the three Enigma LIM domains (Fig. 3, S2, and S3). Tungus is present in the nematode *Caenorhabditis elegans* (Ce_alp-1) and the invertebrate chordate *Branchiostoma floridae* (Bf_123730), but absent from all other species in our study (Fig. S6, S7 and S8).

We found a single Enigma protein in *Nematostella*, *Trichoplax*, *Mnemiopsis*, and *Amphimedon*. We did not find an Enigma in *Drosophila* or in *C. elegans*, but in addition to the three human Enigma proteins, we detected one Enigma in the lophotrochozoan *Capitella teleta* (JGI Capca1|63591). We were unable to recover an Alp from any of the non-bilaterian species, *Drosphila*, or *C. elegans*, but we did find Alp proteins in *Capitella* (JGI Capca1|190169) and *Branchiostoma* (Bf_124330), as well as human.

A previous study, based on the distribution of domains and relationship of a limited set of bilaterian LIM proteins, suggested that a Tungus-like ancestor gave rise to the Alp and Enigma families [22]. However, this hypothesis seems unlikely given the presence of the Enigma family in *Capitella*, as well as in non-bilaterian genomes; all these data were unavailable at the time of the previous study. The presence of the ALP motif throughout the ENIGMA class further contradicts this hypothesis. The most parsimonious explanation given this new data is that an Enigma-like ancestor originated in the stem of the Metazoa and gave rise to the Alp and Tungus families in the stem of the Bilateria (Fig. 2).

### EPLIN class

EPLIN class proteins promote the bundling and stabilization of actin stress fibers and act as scaffolds to associate cell adhesion machinery (specifically, cadherin-catenin complexes) with the cytoskeleton [41]. The mammalian EPLIN gene Lima1 can be found in the cleavage furrow during early embryogenesis (potentially as a recruiter protein) and is also required for cytokinesis [42]. Xirp2 is expressed in skeletal muscle and intercalated discs, where it is required for normal heart development in mice [43].

We identified a highly conserved 22-amino acid motif, which we have named the Eplin Motif, positioned adjacent to the carboxyl-terminus of the EPLIN LIM domain (Fig. 1D, Table S2). In addition to human Lima1 and Xirp2 proteins, we identified this motif-domain combination in a third human protein, Limd2. We also found a single EPLIN class protein with this architecture in each of *Drosophila, Trichoplax, Nematostella*, *Amphimedon*, *Salpingoeca*, *Capsaspora*, as well as three in *Monosiga* (Table S1), which dates the origin of this class to before the last common ancestor of *Capsaspora* and Metazoa (Fig. 2).

The *Amphimedon* EPLIN also contains a troponin-like interaction domain, potentially for binding to either actin or tropomyosin. The *Salpingoeca* EPLIN encodes a SLyX domain that has no known function. One of the *Monosiga* proteins has a carboxyl-terminal cyclic nucleotide binding domain and an EF-hand domain. We were unable to identify an obvious EPLIN in *Mnemiopsis*.

### LASP class

The three vertebrate LASP proteins – Lasp1, Nrap, and Nebl – are closely related to the non-LIM protein Neb. Like Neb, LASP proteins are able to stabilize both F-actin filaments and focal adhesion plaques via nebulin repeats. Nrap is a striated muscle protein involved in myofibril assembly and sarcomere organization. The Nebl gene encodes multiple isoforms, including two that have the characteristic LASP domain architecture and one that has a non-LIM architecture. The latter, also known as Nebulette, encodes over 20 nebulin repeats and no LIM domains. The two LIM domain-containing isoforms (also known as Lasp2) are most highly expressed in the brain as an actin cross-linking structural protein (reviewed in [44]). Lasp1 is the only known nebulin protein to be found in the nucleus as well as the cytoplasm [44], [45].

Human Lasp1 contains a single LIM domain followed by two nebulin repeats and an SH3 domain. Nebl has a similar architecture, but with an additional nebulin repeat, while Nrap contains numerous nebulin repeats and lacks an SH3 domain (Fig. 1E). We identified a single LASP protein with a LIM, two nebulin repeats, and an SH3 domain in *Drosophila*, *Mnemiopsis*, and *Amphimedon*. Three tandemly duplicated proteins with the same architecture were also found in *Nematostella*. No LASP class proteins were found in *Trichoplax*. A single related protein with only one nebulin repeat was identified in the two choanoflagellates and *Capsaspora*. However, the *Monosiga* homolog contained two additional carboxy-terminal SH3 domains, while the *Salpingoeca* homologs contained three. This phylogenetic distribution suggests that the LASP class originated prior to the last common ancestor of *Capsaspora* and Metazoa (Fig. 2).

Domain spacing in all animal LASP proteins besides Nrap is highly conserved. The first nebulin repeat always occurs exactly 67 amino acids from the amino-terminus, while the second one occurs at or near amino acid position 102. Likewise, the LIM domain is always five or six positions from the amino-terminus. Furthermore, the distance between the LIM domain and first nebulin repeat in animals (62 amino acids) is identical to the length of the corresponding interval between the LIM domain and the single nebulin repeat in the *Capsaspora* and *Salpingoeca* LASPs. The spacing in human Nebl is also consistent with this trend. All five of the LASP class proteins in the non-human metazoans in this study contain two rather than three nebulin repeats, suggesting that the domain architecture of Lasp1, rather than Nebl, is the ancestral domain configuration.

Outside of the LASP class, we were unable to find other nebulin repeat-containing proteins in any of the non-human species in this study. This is consistent with previous studies that report only being able to find nebulin repeat-containing proteins in vertebrates and the cephalochordate *Branchiostoma floridae* [46]. This phylogenetic distribution supports the hypothesis that an ancestral LASP gene gave rise to all genes that code for nebulin repeats in metazoan evolution [46]. The rigid spatial requirements on the domains of the LASP proteins might be why there have been so few redeployments of nebulin repeats in the evolution of animals.

### LHX class

LIM homeodomain proteins (LHX) are transcription factors that usually consist of two amino-terminal LIM domains and one carboxyl-terminal homeodomain (Fig. 1F). This class of LIM proteins plays an important role in tissue specification, particularly in the nervous system, where LHX proteins work in combination to determine neuronal fates. This cooperative interaction has been termed the “LIM code” (reviewed in [47]).

In vertebrates, LHX proteins are involved in patterning the head and limbs, and the organogenesis of the forebrain, spinal cord, pituitary, heart, kidneys, eyes, and pancreas (reviewed in [6], [48], [49]). In *Drosophila*, LHX proteins are involved in axon guidance, patterning, and muscle formation (reviewed in [50]). LHX gene expression has been observed in presumptive neural territories during *Nematostella* development and in the photoreceptor ring of *Amphimedon* [23].

Previous studies have suggested that LHX proteins are metazoan innovations (e.g., [23]). Consistent with these studies, we recovered LHX proteins from all of the metazoans in our study, whereas none were found in the three non-metazoan proteomes. This phylogenetic distribution suggests that LHX proteins originated at the stem of the Metazoa (Fig. 2). In total, we recovered three *Amphimedon*, four *Mnmeiopsis*, four *Trichoplax*, six *Nematostella*, six *Drosophila*, and 12 human LHX proteins (Table S1). *Trichoplax* has two additional LHX proteins that are absent from JGI's proteome version 1.0, but were described by Srivavstava and coauthors, making for a total of six LHX proteins [23].

### LMO class

Unlike LHX transcription factors, nuclear LMO proteins lack a DNA-binding homeodomain (Fig. 1G). However, the two LIM domains of the LMO proteins each form a corresponding clade with the two LIM domains of LHX proteins, suggesting that these two classes are sister groups (Fig. 3, S2 and S3).

LMO proteins regulate gene expression by binding transcription factors and other nuclear proteins. For example, in many cell types, “LIM Only” (LMO) proteins are co-expressed with LHX proteins and are thought to play a role in antagonizing selected LHX combinations (reviewed in [51]). In this way, LMO proteins negatively regulate the “LIM code.”

In addition to the four human LMO proteins and two *Drosophila* LMO proteins, we identified three LMO proteins in *Nematostella* and one protein in *Trichoplax* (Table S1). No LMO proteins were recovered from *Capsaspora*, *Monosiga*, *Salpingoeca*, *Mnemiopsis*, or *Amphimedon*. Given the phylogenetic distribution of these lineages and the corresponding relationship of the two LIM domains of LMO and LHX in our tree (Fig. 3, S2, and S3), the most parsimonious explanation is that an ancestral LHX-like gene lost its homeobox somewhere in the stem of the ParaHoxozoa, thereby forming the LMO class (Fig. 2).

### LIMK class

LIMK proteins are serine/threonine kinases that inhibit actin disassembly by phosphorylating cofilin proteins (reviewed in [4], [52]). Through this interaction, LIMK proteins regulate cell spreading, motility, growth, and cytokinesis. Moreover, LIMK proteins localize to focal adhesions, where they catalyze signaling cascades, or they can be shuttled to the nucleus where they regulate transcription [52]. Homo-dimerization of LIMK proteins may inhibit kinase activity or, in complex with a mediator, can enhance kinase activity (reviewed in [4]).

LIMK proteins contain two amino-terminal LIM domains, a PDZ domain, and a kinase domain (Fig. 1H). In addition to the human LIMK1 and LIMK2 proteins, we identified single LIMKs in *Drosophila*, *Nematostella*, and *Amphimedon*. No LIM domains from *Trichoplax*, *Mnemiopsis*, *Salpingoeca*, or *Monosiga* are present in the two clades that comprise the LIMK LIM domains (Fig. 3, S2 and S3). Furthermore, we were unable to identify any proteins with both a kinase domain and a LIM domain from these four species. LIMK appears to be absent from these species.

*Capsaspora* has three proteins that have both kinase and LIM domains. We chose to exclude two of the *Capsaspora* proteins (Co_06515T0 and Co_08582T0) from the LIMK class. These two have atypical domain architectures, which lack PDZ domains; in addition, each contains more than two LIM domains, none of which share phylogenetic affinity with the *bona fide* LIMK LIM domains. The other (Co_05847T0) has a typical LIMK domain architecture, but also contains an additional TFIIA domain (Pfam PF03153). Although the first LIM of this protein is highly divergent, the second LIM is phylogenetically related to the second LIM of the metazoan LIMK proteins (Fig. 3, S2 and S3). We have classified this as a true LIMK and as such, date the origin of this class prior to the last common ancestor of animals and Capsaspora (Fig. 2).

### LMO7 class

The canonical LMO7 proteins consist of a CH domain, a PDZ domain, and a single LIM domain (Fig. 1I). The mammalian Lmo7 protein is involved in actin polymerization and stabilizing F-actin [53], [54]. It localizes to focal adhesions, but in response to mechanical stress, can shuttle to the nucleus, where it is a potent transcriptional regulator [55].

We found related single LIM proteins in both *Drosophila* and *Nematostella*. The *Drosophila* protein, which lacks both PDZ and CH domains (Dm_CG31534), had previously been designated as an LMO7 [22]. In *Nematostella*, we recovered a single protein (Nv_216756) with a LIM domain and a degraded CH, but no PDZ. Interestingly, we identified LMO7 proteins, each with a single PDZ and CH domain, in *Amphimedon* and *Mnemiopsis*, but did not find any LMO7 proteins in the non-metazoan species. The presence of these proteins in the two earliest animal lineages suggests that LMO7 originated at the stem of the Metazoa (Fig. 2).

According to our phylogenetic analysis, the human Limch1 and Znf185 proteins are closely related to human Lmo7 (Fig. S4 and S5). Limch1 contains a single LIM domain and a CH domain, but lacks the PDZ domain. Znf185 lacks both the PDZ and CH domain but unlike other LMO7 class protein, has an amino-terminal domain called an actin-targeting domain (ATD), which is required for Znf185 to localize to actin-regulated structures [56]. In our multi-species tree (Fig. 3, S2 and S3), Limch1 and Znf185 form a clade with human Lmo7 and the *Drosophila* Lmo7 within the larger LMO7 clade suggesting that these proteins are likely the product of bilaterian-specific gene duplications.

### MICAL class

MICAL is a single LIM domain-containing class consisting of the Mical and Mical-like families. Proteins of the Mical family are involved in destabilizing actin for neuronal growth and axon guidance during embryogenesis. They are expressed throughout adulthood in lung, brain, heart, thymus, and particularly in neuronal and muscular tissues. Mical-like proteins are involved in vesicular trafficking and the recycling of tight junction components (reviewed in [57]).

In addition to a single LIM domain, MICAL class proteins have an actin-binding calponin homology (CH) domain and a highly conserved carboxyl-terminal region, represented by PFAM model DUF3585 (Pfam PF12130; Fig. 1J). The Mical family is distinguished from the Mical-like family by an additional amino-terminal catalytic FAD-binding/oxidoreductase domain, which is required for Mical to bind F-actin [57]. We found that the Pfam FAD-binding HMM (Pfam PF01494.12) was not sensitive enough to identify all FAD-binding domains of the Mical family. Furthermore, we found that the entire region from the amino-terminus to the CH domain, which incudes the FAD-binding domain in MICAL proteins, is highly conserved across Metazoa. Therefore, we constructed two HMMs to represent the regions surrounding the PFAM-predicted FAD-binding domain in Mical family proteins (Fig. S9).

We were unable to identify any MICAL class proteins from the non-animal genomes in this study. On the other hand, both Mical and Mical-like proteins were found in each animal we investigated except for *Trichoplax*, which encoded a single Mical protein. This phylogenetic distribution suggests that both the MICAL class and the Mical and Mical-like families were established at the metazoan stem (Fig. 2). In an attempt to better resolve the relationships between the ENIGMA, LIMK, LMO7, and MICAL classes, we performed a phylogenetic analysis on the PDZ and CH domains of these proteins (data not shown). Unfortunately, the results of this analysis were inconclusive and were, therefore, not included.

### PXN class

Like ABLIM, PXN (Paxillin) is a class of focal adhesion scaffolding and integrin-mediated signaling proteins [58]. PXN proteins encode four carboxyl-terminal LIM domains, which localize these proteins to focal adhesions. They also encode one or more amino-terminal LD motifs, which are short leucine-aspartate-rich regions that have the consensus sequence LDxLLxxL (Fig. 1K). These LD motifs are required for interaction with many other proteins [59].

When phosphorylated, PXNs can recruit complexes of proteins to focal adhesions and regulate Rho GTPase signaling to effect cell adhesion, spreading, motility, and survival (reviewed in [60], [61]). In human cells, the Tgfb1i1 and Pxn proteins have been shown to shuttle between the cytoplasm and nucleus, where they serve as nuclear receptor co-activators [58], [62].

PXNs can be found in both fungi and amoebae and, as such, are an ancient class of LIM protein (Fig. 2) [60]. We found a single PXN in each genome we surveyed except for human, which encodes three (Table S1). We identified LD motifs in the PXNs of all animals and *Capsaspora*, but not in either of the choanoflagellates. In addition to a true PXN protein, *Capsaspora* has an additional PXN-like protein with four divergent PXN LIM domains as well as a Rap-GAP domain, but no identifiable LD motifs (Co_06505T0 in Table S1).

### PINCH class

PINCH (sometimes called LIMS) proteins are adapters responsible for focal adhesion assembly and linking integrins to multiple signaling pathways (reviewed in [61], [63], [64]). PINCH proteins complex with integrins at muscle attachment sites [65] and also have been shown to shuttle to the nucleus in Schwann cells and neurons [66].

PINCH proteins contain five tandem LIM domains (Fig. 1L). We also identified a highly conserved twelve amino acid PINCH motif. This leucine-rich motif occurs immediately adjacent to the C-terminal side of the five LIM domains (Table S2). We found a single PINCH protein in *Drosophila*, *Nematostella*, *Trichoplax*, and *Amphimedon*. The *Mnemiopsis* genome encodes two PINCH proteins and the human genome encodes three (Table S1). No PINCH proteins were observed in either of the choanoflagellates, but a PINCH protein exists in *Capsaspora*, which sets the origin of the PINCH class prior to the last common ancestor of metazoans and Capsaspora (Fig. 2).

### TES Class

The TES class consists of the Tes, Etes, and Fhl families. The PET domain is a highly conserved putative protein-protein interaction domain [67] that is specific to metazoans and choanoflagellates. The domain is characteristic of Tes and Etes families. The Fhl family originated recently in evolution and is characterized by the loss of the PET domain.

We identified two novel motifs in TES class proteins that we call TMA1 and TMA2 (Table S2). These motifs always occur to the amino-terminal region of the PET domain (Table S1). Seven of the TES class proteins have both of these motifs, which, in all cases, are separated by 17 or 18 amino acids. This suggests that they are part of a larger ∼60 amino acid motif. 18 of the 28 proteins that make up the Tes and Etes families have at least one of these motifs (Table S1). In the human Lmcd1 protein, the region corresponding to the TMA2 motif is reported to bind the GATA6 transcription factor [68], suggesting that this motif is somehow related with transcriptional activities. We did not detect the motif in any of the FHL proteins. The presence of this motif in Tes family proteins of *Monosiga* suggests that this motif was one of the founding components of the class.

#### Tes family

Proteins of the Tes family are characterized by an amino-terminal PET domain and two to three carboxyl-terminal LIM domains (Fig. 1M). The PET domain is capable of binding its own LIM domains and subsequently altering its set of binding partners; this, in turn, regulates its cellular localization [69]. Human Tes localizes to focal adhesions and is involved in cell spreading [70]. It has been shown to be present in the nucleus and is potentially involved in shuttling, similar to other LIM proteins [71].

*Drosophila* Prickle and Human Prickle1 and Prickle2 are classically described as core components in the non-canonical Wnt planar cell polarity (PCP) pathway. In this pathway, these proteins antagonize Dsh on the proximal side of the cell, inducing a distal Fz-Dsh complex and establishing cell polarity (reviewed in [72]).

We identified Tes family proteins in all species surveyed except for *Capsaspora.* This phylogenetic distribution suggests that Tes proteins originated just prior to the last common ancestor of chonanoflagellates and animals (Fig. 2).

#### Etes family

We have designated TES class proteins that contain a PET domain and six LIM domains as the Etes (for “Extended testin”) family (Fig. 1M). We recovered one Etes family protein from both *Drosophila* and *Amphimedon* and two from *Nematostella* (Table S1). There is limited literature describing the Etes proteins from these three species. However, the *C. elegans* ortholog, lim-8, is a component of the focal adhesion complex at muscle wall sarcomeres [73], and is expressed in neurons, depressor muscles, and other tissues [74]. The presence of an Etes protein in *Amphimedon* but not in any of the non-metazoans suggests that this family originated in the stem lineage of Metazoa (Fig. 2).

#### Fhl family

Fhl (for “Four and a half LIM”) proteins contain four LIM domains and a LIM-like amino-terminal zinc-finger domain (the “half LIM”; Fig. 1M). These five domains share corresponding homology with the terminal five LIM domains of *Nematostella* and *Drosophila* Etes family proteins. Humans lack an Etes family protein and are the only species in our study with Fhl proteins. The most parsimonious explanation for this data is that an ancestral Etes-like protein lost its PET domain somewhere in the lineage to humans after it split from *Drosophila* (Fig. 2).

Members of the human Fhl (Four and a half LIMs) family are highly expressed in striated muscle, osteoblasts, and testes, where they have documented interactions with more than 50 other proteins [9], [75]. They are involved in integrin-mediated, Notch, TGF-β, and Rho signaling, co-transcriptional activation and repression, cell differentiation, cytoskeletal remodeling, and mechanical stress response [6], [9], [75]. Their involvement in skeletal/cardiac myopathies and metastatic cancers is well-characterized [75].

### ZYX class

ZYX (Zyxin) class proteins act as adapter proteins that facilitate the assembly of protein complexes at focal adhesions and take part in traffic to and from the nucleus (reviewed in [76]). ZYX proteins are characterized by three closely spaced carboxyl-terminal LIM domains that are required for localization to focal adhesions and adherens junctions (reviewed in [77], [78]; Fig. 1N). The amino-terminal region of ZYX proteins are highly variable, leading to a diverse set of binding partners within the class [77]. ZYXs are implicated in cell fate determination, cell motility, oncogenesis, and cell growth ([76], [77]). Recent work has shown that ZYXs also play a role in microRNA silencing and telomere protection [79], [80].

We recovered seven ZYX proteins from human, three from *Drosophila*, two from *Nematostella*, and one each from *Amphimedon* and *Mnemiopsis* (Table S1). We were not able to identify any ZYX proteins in the *Trichoplax* or non-animal genomes. The phylogenetic distribution of the ZYX class suggests that this class arose in the stem of the Metazoa (Fig. 2).

We identified a leucine-rich amino-terminal motif in *Drosophila* Jub, five of the seven human ZYXs, and one of the *Nematostella* ZYXs. In the human LPP protein, this motif overlaps with a functional leucine-rich nuclear export signal. We used the NetNES algorithm to predict putative nuclear export signals in the non-bilaterian ZYXs and found one overlapping with this same motif in the *Nematostella* ZYX protein [81]. In addition, we also found putative nuclear export signals in the *Mnemiopsis* and *Amphimedon* ZYXs despite the lack of the motif in these proteins, suggesting that nuclear shuttling is an ancestral trait of this class.

### Unclassified Proteins

Fifty-nine proteins did not meet the criteria required to be included in one of the LIM classes. Depending on the complexity of domain architecture in a class, our criteria included a reasonable subset of these requirements: (1) conservation of LIM quantity, (2) phylogenetic affinity of LIM domains with the LIM domains of human proteins within the class, (3) presence of non-LIM domains and/or motifs that are characteristic of the group, and (4) correct order of LIM and non-LIM domains and/or motifs.

Most of these 59 proteins include domain architectures not seen in any of the described classes. Many of these proteins could not be categorized since they represent lineage-specific innovations that no longer fit the criteria for membership to an existing class. Others may be the result of erroneous gene predictions in the genomic region of a classifiable LIM gene. However, we were able to identify a group of possibly related proteins from *Drosophila, Trichoplax*, and *Amphimedon* (Dm_Rassf, Aq_215865, Ta_55975) with the conserved architecture of an amino-terminal LIM domain and a carboxy-terminal RasGTP association domain (Pfam PF00788). Further phylogenetic analysis is needed to assess whether this group represents a novel class of metazoan LIM proteins.

It is worth noting that 37 of the 59 unclassified LIM proteins are from the three non-metazoan species. This is not surprising, since the non-metazoan species have had a longer stretch of independent evolution and have experienced much different selective pressures than metazoans, especially in terms of their cell surface environments.

We also note here that this study did not characterize two of the 73 described human LIM genes, SCEL and LIMS3L. These genes have been included in the “Unclassified” section of Table S1 for completeness.

## Discussion

### LIM domains are building blocks of subcellular complexity

LIM domain-containing proteins have a range of binding partners and are considered “molecular adapters” because of their ability to assemble proteins that would otherwise be unable to interact directly. The binding flexibility of the LIM domain is also used for autoregulation, as well as for the combinatorial or direct regulation of other proteins. Most LIM proteins serve in cytoskeletal complexes but can also translocate to the nucleus to regulate transcription. In this way, they are vital for communicating extracellular signals between the surface of a cell and the nucleus. This dual localization makes LIM proteins important for the modulation of cell motility, structure, and division.

In this study, we have identified 265 LIM domain-containing proteins from nine proteomes. We divided this LIM complement up into 14 classes. Our classification relied on both phylogenetic analyses of LIM domains, as well as domain and motif architecture; in one case, phylogenetic analyses of non-LIM domains were also applied. For each class and family, we have provided plausible estimates of origin, which are summarized in Figure 2.

### New LIM domain architectures in the metazoan stem

**Figure 4:**
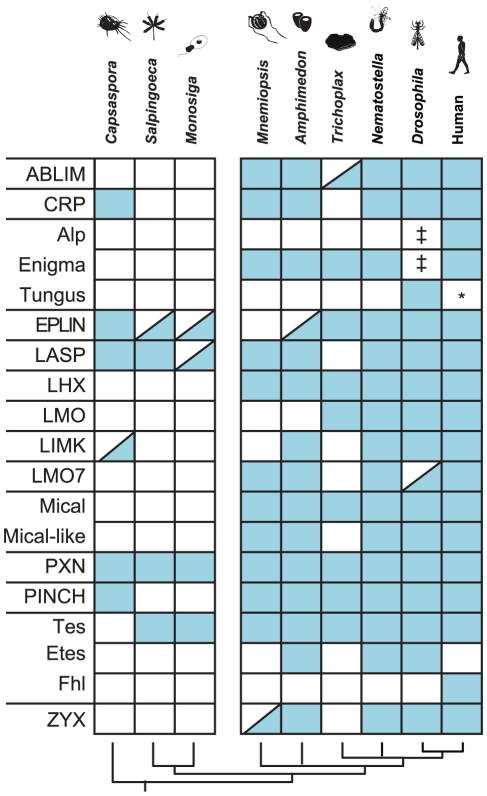
Presence and absence of LIM classes in our sampled species. The left column represents classes (designations written in all caps) or families (designations written in title case and clustered by class). There is a break between columns representing non-metazoans and metazoans to highlight the small number of classes and families present in the non-metazoans. Blue squares represent presence of a particular class or family (row) in a particular species (column). A half-blue square indicates some uncertainty as to the whether or not a particular class or family is present. Notes on half-blue squares: (a) both *Trichoplax* ABLIM proteins lack a VHP domain; (b) the *Capsaspora* LIMK protein contains an extra TFIIA domain; (c) the *Amphimedon*, *Monosiga*, and *Salpingoeca* EPLIN proteins contain additional domains besides the EPLIN motif and LIM domain; (d) the *Monosiga* LASP protein contains an additional PH domain; (e) the *Drosophila* LMO7 contains only an LMO7-like LIM domain, but lacks a CH domain and a PDZ domain; (f) the *Mnemiopsis* ZYX protein contains extra DSL domains. ‡ Alp and Enigma are absent from *Drosophila* but they are both present in another protostome *Capitella telata*. * Tungus is absent from *Homo sapiens*, however we positively identified a Tungus protein in another deuterostome *Branchiostoma floridae*.

Novel combinations of protein domains have been produced by domain fusion and recombination events throughout evolution. These events (and their fixation) are somewhat rare, but have been shown to be relatively constant, with bursts of increased domain promiscuity occasionally occurring between various ancestral nodes [82]. Our analysis suggests that an impressive burst of domain promiscuity occurred in the stem lineage of the Metazoa (Fig. 2 and 4). This LIM architecture expansion is especially remarkable, considering how important adaptations to cell-surface signaling would be to a lineage in transition to a multicellular lifestyle. The shift of a cell's surface substrate from an external environment to one consisting primarily of adjacent cells and a protein matrix provided the niches necessary for these new LIM classes to become fixed in the metazoan lineage. The organisms with a larger array of these proteins most likely had a better chance of inventing new cell types.

Similarly, *Trichoplax* appears to have lost the LASP, LMO7, LIMK, ZYX and CRP classes. If it is true, as most phylogenetic (reviewed in [83]) and morphological [84] evidence suggests, that *Trichoplax* has secondarily lost musculature and a traditional nervous system, it is perhaps not surprising that this species would have lost these classes of proteins, which serve a prominent role in the formation of these tissues. Moreover, it is not inconceivable that these losses might have contributed to a reduction of the cell types necessary for the maintenance of these systems in the *Trichoplax* lineage.

### Conclusion

Our analysis and classification of the LIM superclass has revealed a pattern of expansion consistent with these proteins playing a major role in the origin of animal multicellularity. The increasing availability of genome-scale sequence data (especially from invertebrate metazoans and close outgroups) will continue to further our understanding of the history of the LIM superclass, allowing for a more precise chronicle of the evolution of the individual LIM classes and families. Furthermore, because human LIM proteins are implicated in diseases as diverse as leukemia, epilepsy, cardiomyopathy, osteoporosis, and muscular dystrophy, understanding the evolutionary history of this superclass can help translational researchers with the identification of medically relevant sequence motifs, the determination of appropriate model species, and the proper association of findings from model systems to human homologs [11], [12], [13], [14], [85], [86].

## Methods

### Sequences

The filtered protein models for *Nematostella* v. 1.0 [87], *Trichoplax* v. 1.0 [88] and *Monosiga* v. 1.0 [89] were downloaded from each species' Joint Genome Institute (JGI) genome website. The *Amphimedon* predicted proteome was downloaded from the link provided in the genome paper (ftp://ftp.jgi-psf.org/pub/JGI_data/Amphimedon_queenslandica/assembly/) [90]. Protein sequences for *Capsaspora* and *Salpingoeca* were downloaded from the Origins of Multicellularity Sequencing Project, Broad Institute of Harvard and MIT (see http://www.broadinstitute.org) in March 2011. The *Drosophila* v. 3.0 proteome was downloaded from the FlyBase Web site [91]. Human protein sequences were downloaded from the National Center for Biotechnology Information's RefSeq ftp site in July 2009. As part of our *Mnemiopsis* sequencing effort, we generated protein-coding gene models using a combination of Fgenesh [92], PASA [93], and EvidenceModeler [94]. The *Mnemiopsis* proteins used in this study are publicly available in GenBank. GenBank accession numbers for all *Mnemiopsis* sequences used in this study can be found in Table S1.

For convenience, we have adopted a simplified naming convention to refer to sequences. For all sequences, the first two characters refer to the genus and species followed by an underscore. For human and *Drosophila* sequences the rest of the name is the Entrez gene symbol or the FlyBase name, respectively (e.g., human gi|5453710|ref|NP_006139.1| is named Hs_LASP1 and *Drosophila* FBpp0075109 is named Dm_Lasp). In the case of human sequences with more than one isoform, the Entrez gene symbol is followed by a hyphen and the number or letter of the isoform as it appears in RefSeq. In the case of genomes sequenced by the Joint Genome Institute, the JGI ID follows the underscore (e.g., jgi|Nemve1|178184|estExt_GenewiseH_1.C_50530 is named Nv_178184). For *Amphimedon* sequences, we used the first number in the sequence header (e.g., Aqu1.224097|PACid:15722625 is named Aq_224097). For *Salpingoeca* and *Capsaspora*, we use the complete gene model ID that was assigned by the Origins of Multicellularity Sequencing project. Similarly, we used the *Mnemiopsis* gene model IDs that our group generated as part of the *Mnemoipsis* genome project. We refer to the LIM domains within these sequences in amino to carboxyl order (e.g., Dm_Lmpt.A corresponds with the most amino terminal LIM domain found in the Dm_Lmpt protein).

### Alignment

We used the LIM HMM (Pfam PF00412.15) from the Pfam protein domain database [25], [26] and the hmmsearch program from the HMMER suite v. 3.0b to recover all LIM domain sequences from each of the nine proteomes. We aligned LIM domains to the LIM HMM using the output of hmmsearch. The hmmsearch program was run using its default settings. The carboxyl-terminus of the LIM domain is quite variable, which makes it difficult for an HMM-based domain detection method like hmmsearch to identify this region of the domain. Consequently, there are carboxyl-terminal gaps in 528 of the 645 LIM domains that we recovered. In about 10% of our sequences, the method failed to detect even the ultra-conserved cysteine at position 50 and the highly conserved residue at position 53 (usually cysteine, aspartic acid, or histidine) of the canonical LIM domain. However, given the vast evolutionary distance between the sampled taxa, these variable regions are not likely to be phylogenetically informative. Therefore, we did not replace this missing data.

For human and *Drosophila* genes with alternatively spliced transcripts, we selected a single representative isoform. We discarded proteins with domains that were highly truncated or had very poor sequence conservation. These sequences represented zinc fingers that were mispredicted as LIM domains. In one case, (Ta_20314) a zinc finger made it into our data set and trees, but was later removed after we performed more detailed analyses. For each domain sequence in our main dataset, all characters predicted as insertions within the HMM (represented as lowercase letters) were removed. We added all individually processed domains to a single file to construct our nine-species alignment (Fig. S1).

### Phylogenetics

We used maximum likelihood (ML) and Bayesian methods in a likelihood framework to construct two phylogenetic trees. We generated one tree (Fig. 3, S2 and S3) from the complete nine-species alignment (Fig. S1) and a second (Fig. S4 and S5) from an alignment consisting of only the human subset of sequences. We ran ProtTest v2.4 [95] to determine that the LG model with gamma distribution of rates and invariant site categories was the most appropriate model to evaluate trees. For each alignment, we conducted two independent maximum likelihood searches using RAxML v.7.2.8a [96]: one with 25 random starting trees with the following command line (raxmlHPC-MPI -f d -m PROTGAMMAILG -s input.phy -#25 -d –k), and another with 25 parsimony starting trees (raxmlHPC-MPI -f d -m PROTGAMMAILG -s input.phy -#25 -k).

We used MrBayes v. 3.1.2 to construct Bayesian trees for each dataset [97]. Because MrBayes does not support the LG model of evolution and no other models received an AIC weight greater than 0.0001, we ran two independent 500,0000-generation runs of five chains with the related WAG model [98] for each alignment with the following execution block (prset aamodelpr = fixed(wag); lset rates = Invgamma; mcmp mcmcdiagn = no nruns = 1 ngen = 5000000 printfreq = 5000 samplefreq = 500 nchains = 5 savebrlens = yes; mcmc;). All runs were found to be asymptotic before the relative burn-in fraction of 0.25. We computed likelihood scores for all trees using the LG matrix in PHYML v3.0 [99] with the following command (phyml -i 01-Input.phy -c 4 -m LG -a e -o lr -f d -u 01-Input.tre -v e -d aa -b 0 -s NNI). We then chose the tree with the highest likelihood from all 50 ML searches and both Bayesian trees (Fig. 3, S2 and S3). Support for clades was assessed with 100 bootstrap replicates with the following command (raxmlHPC-MPI -m PROTGAMMAILG -s 01-Input.phy -N 100 -n 100BS –k).

### Classification of LIM Domain Sequences

Because bootstrap support for the main dataset phylogeny was poor, we used a consensus approach to identify clades that were recovered independently in both the main dataset and the human-specific subset. We created a strict consensus cladogram of human taxa using Figure S5 and a pruned version of Figure S3. We rooted this tree at the midpoint to create 38 basal clades of human LIM domains. For convenience, we call these clades “homology groups” and the human LIM domains within them “members” of these homology groups.

Beginning with the nine-species tree (Fig. S3), we used a nearest neighbor approach to assign non-human LIM domains to homology groups. For each non-human leaf, we identified the most recent common node shared with a human leaf. If all human leaves descending from that common node belong to the same homology group, the leaf was placed in that homology group. If the most recent common node belonged to multiple homology groups, the leaf was declared unclassifiable. The homology group to which each LIM domain belongs is listed in Table S3, along with the class and position of the conserved LIM domain most common in that group. In Figure 2 and S2 the alternating branch colorings distinguish between different homology groups.

### Domain Architecture Description

We used the HMMER program hmmscan and Pfam v 24.0 to detect other domains in all the proteins of our main dataset [25], [26]. The hmmscan program was run using its default settings. Predictions with an independent *E*-value above 0.05 were excluded. In the case of overlapping domain envelopes, the prediction with the lowest independent *E*-value was selected. Predictions removed in this manner were checked individually.

### Motif Discovery

Low complexity regions were masked out of all proteins in the main dataset using TANTAN v. 3 [100], as were Pfam-predicted domains with an *E*-value below 0.05. The TANTAN program was run using its default settings. We then ran the MEME motif discovery program iteratively, searching for a single motif in at least four proteins with the following command line (meme -minsites 4 -p 6 -maxsize 1000000 INPUT_FILE) [27]. All discovered motifs were masked before running additional iterations. This process was repeated until motifs with *E*-values greater than 0.01 were reported. The results of these analyses are shown in Table S2.

We ran MEME on an unmasked version of the LIM proteins to identify instances of existing motifs that may have been masked. We did not consider new motifs from this unmasked alignment, but in some cases extended existing motifs. All modifications stemming from this unmasked analysis are indicated in Table S2.

### MICAL Hidden Markov Models

We identified multiple motifs in the highly conserved N-terminus in MICAL proteins in the motif discovery analysis. We aligned the proteins containing these motifs using MUSCLE v3.8.31 [101]. We then used HMMER's hmmbuild program to create HMMs (Fig. S9) for the regions N-terminal and C-terminal to the envelope of the FAD-binding domain predicted by Pfam (Pfam PF01494). The default settings for hmmbuild were used for this analysis.

### ENIGMA Class Phylogenetic Analyses

To more precisely date the origin of the Alp and Tungus families, we expanded our main dataset to include PDZ- and LIM-containing proteins from the following additional bilaterian proteomes: *Caenorhabditis elegans* WS219 (from Wormbase), *Capitella teleta* v1.0 (from JGI), *Lottia gigantea* v1.0 (from JGI), *Saccoglossus kowalevskii* (from RefSeq), *Strongylocentrotus purpuratus* (from SpBase), *Branchiostoma floridae* v2.0 (from JGI), *Ciona intestinalis* v2.0 (from JGI), *Gallus gallus* (from Refseq), *Danio rerio* (from Refseq) [102], [103], [104], [105], [106]. We also BLASTed Dm_Tungus, Hs_PDLIM3, and Hs_PDLIM7 against the *C. elegans*, *Capitella*, and *Branchiostoma* genomes to ensure that no unpredicted genes were omitted from these species (see Table S1 for accessions).

We used hmmscan (as described above) to identify proteins containing both PDZ and LIM domains in each additional species [26]. We constructed a new multiple alignment, which included the LIM domains from these sequences and the LIM domains of the PDZ-LIM proteins from our nine-species dataset (Fig. S6). We then used the same strategy employed for the LIM trees above on this alignment and generated a tree (Fig. S6 and S7).

### ZASP and ALP Motifs

We searched for the Zasp Motif in all proteins in the main dataset using the corresponding SMART HMM (SM00735; Table S2) [40]. The Alp motif was recovered in the motif analysis (Table S2), but for greater resolution, we created a HMM from the multiple sequence alignment curated by te Vethuis *et al.* [38]. We searched for this motif in the full dataset combined with the *Brianchiostoma*, *Capitella* and *C. elegans* PDZ-LIM models identified above with the following command (hmmsearch –max –incE 10 AM_MOTIF.hmm Input.fa). The results are reported in Table S2.

### Nebulin Repeat Analysis

In order to increase our confidence that nebulin repeats are specific to the Lasp family in non-bilaterians, we performed the following analysis. First, we ran Augustus and HMMgene on each of the non-bilaterian genomes in our study [107], [108]. Next, we translated these genomes in six frames. Finally, we searched these hypothetical proteomes, along with the published proteomes, for nebulin repeats using hmmscan.

### LIM Protein Classification Criteria

We classified the human LIM proteins into 14 classes based on sequence similarity and domain architectures. Our phylogenetic analysis validates these groups. We assigned non-human LIM proteins to these groups if they (1) shared the same number of LIM domains as human members of the class, (2) shared the same complement of LIM homology groups as human members of the class; (3) shared the conserved order of LIM domains found in human members of the class, and (4) shared non-LIM domains, motifs, and arrangement of these architectural features distinctive of the class.

### Missing Domains and LIM Classes

To be certain that species-specific class absences of classes were not a result of errors in published proteomes, we performed the following analysis. First, we used Fgenesh [92] to predict proteins *de novo* in the *Amphimedon* and *Salpingoeca* genomes and created a multiple alignment of the LIM domains found in these models. To this alignment, we added LIM domains found in JGI unfiltered protein models for *Nematostella*, *Trichoplax*, and *Monosiga*. After removing duplicates from our main analysis, we repeated the full phylogenetic and LIM domain classification analyses to place these LIM domains into homology groups. For each species, we looked for homology groups not present for that species in the main dataset. We recovered one *Amphimedon* protein in this analysis and submitted it to Genbank (GenBank JN615191).

For some JGI proteins, we found alternative models with more conserved domain architectures than the filtered model following phylogenetic characterization of the LIM domains. When a superior model was discovered, that model (and not the filtered model) was entered into Table S1. In almost all cases, the LIM sequences in these new models are either identical to or more complete than those from the filtered models used in the phylogenetic analysis. Where they do exist, discrepancies between LIM domain sequences from different models are noted in Table S1.

## Supporting Information

Figure S1 **Multiple sequence alignment of LIM domain.** This alignment includes LIM domains from nine species. The alignment is in FASTA format. Due to the automatic nature of our LIM identification, many of the LIM domains are incomplete, especially at the carboxyl-terminus. This is discussed in more detail in the Methods.
(FA) Click here for additional data file.

Figure S2 **LIM domain tree.** Midpoint rooted phylogram of LIM domain phylogeny (maximum likelihood). Alternating blue and grey coloring delineates homology groups; black regions are unclassified. Conserved LIM group labels appear within the upper edge of a clade. See Figure 2 for more details on homology groups and tree labeling. See Table S1 for details on individual sequences. See Table S1 for the corresponding alignment. Node values denote the percentage of 100 bootstrap replicates recovered for that particular bipartition.
(PDF)

Figure S3 **LIM domain tree in Newick format.** Newick version of Figure S2. This file can be opened and manipulated in tree-viewing software like Figtree or Treeview.
(TRE)

Figure S4 **Human LIM domain tree.** Midpoint rooted phylogram of human LIM domain phylogeny (maximum likelihood). See Table S1 for details on individual sequences. Node values denote the percentage of 100 bootstrap replicates recovered for that particular bipartition.
(PDF)

Figure S5 **Human LIM domain tree in Newick format.** Newick version of Figure S4. This file can be opened and manipulated in tree-viewing software like Figtree or Treeview.
(TRE)

Figure S6 **Multiple sequence alignment of ENIGMA, LIMK, and LMO7 LIM domains.** This alignment contains the subset of sequences from Figure S1 that were found in proteins classified as ENIGMA, LIMK, or LMO7. LIM domain sequences taken from proteins that contain PDZ and LIM domains from *Branchiostoma floridae*, *Caenorhabditis elegans*, *Capitella teleta*, *Ciona intestinalis*, *Danio rerio*, *Gallus gallus*, *Lottia gigantea*, *Saccoglossus kowalevskii*, and *Strongylocentrotus purpuratus* were added to this alignment. The alignment is in FASTA format.
(FA) Click here for additional data file.

Figure S7 **LIM domain tree from ENIGMA, LIMK, and LMO7 class proteins.** Midpoint rooted phylogram of ENIGMA, LIMK, and LMO7 class LIM domain phylogeny (maximum likelihood). See Table S1 for details on individual sequences. Node values denote the percentage of 100 bootstrap replicates recovered for that particular bipartition.
(PDF)

Figure S8 **LIM domain tree in Newick format from ENIGMA, LIMK, and LMO7 class proteins.** Newick version of Figure S7. This file can be opened and manipulated in tree-viewing software like Figtree or Treeview.
(TRE)

Figure S9 **Hidden Markov models for conserved MICAL amino-terminus region.** This RAR file contains two HMMs that span from the MICAL amino-terminus to the CH domain. One is amino-terminal to the FAD_Binding3 Pfam domain; the other is carboxyl-terminal. The files are in HMMER format.
(RAR)

Table S1 **Classification of LIM proteins.** Species, accession numbers, and domain architectures are provided for each LIM protein in our analysis. Blue and grey columns indicate the amino acid position of a particular domain or motif as well as the E-Value from hmmsearch, in the case of domains, and MEME, in the case of motifs. Blank blue and grey columns indicate that the particular domain or motif was not found. A single asterisk indicates a feature that was not identified in the original protein sequence, but is present in alternative protein models. A note at the end of the row describes the alternative model associated with the asterisk. A double asterisk refers to a class-level note listed at the top of the class. Domains in red indicate domains that are not typical of the class.
(XLS)

Table S2 **Motifs of LIM proteins.** Each motif includes a MEME score in parenthesis next to the motif name, as well as a regular expression that defines the motif. We manually adjusted regular expressions in some cases to ensure that they matched all sequences identified by MEME. Residues in red represent those that were discovered by MEME using an unmasked version of the LIM proteins. Notes at the bottom of a section indicate other proteins where this motif was identified in the unmasked version of the MEME analysis. In the case of motifs missed by MEME, but discovered using our manually adjusted regular expression, the term “Regex” appears in the E-Value column.
(XLS)

Table S3 **LIM domain homology groups.** We created 38 LIM domain homology groups based on concordant clades from a strict consensus of our human LIM domain tree (Fig. S3) and a pruned version of our nine-species LIM domain tree (Fig. S5). We assigned non-human LIM domains to these homology groups based on a nearest-neighbor analysis. Letters following the protein name represent the position of the LIM domain within the particular protein (e.g., Hs_ABLIM2.B refers to the second LIM domain in the Hs_ABLIM protein).
(XLS)
