# Supplementary figures and images for: The Diversification of the LIM Superclass at the Base of the Metazoa Increased Subcellular Complexity and Promoted Multicellular Specialization
Source: PLoS One. 2012 Mar 15;7(3):e33261. doi: 10.1371/journal.pone.0033261 (PMC3305314; doi:10.1371/journal.pone.0033261)

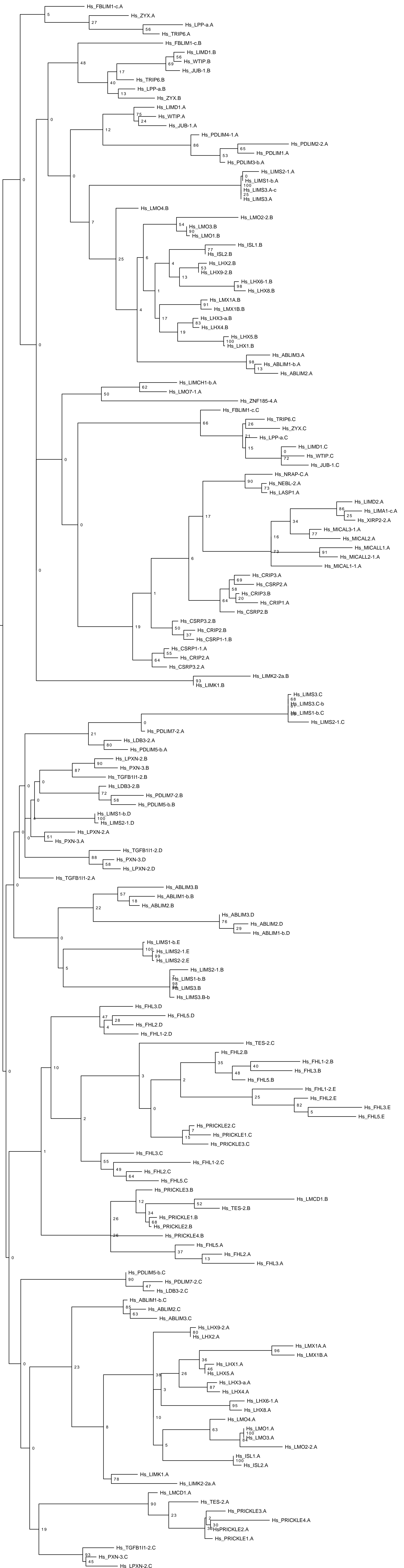

Supplement: Figure S4 — Human LIM domain tree. Midpoint rooted phylogram of human LIM domain phylogeny (maximum likelihood). See Table S1 for details on individual sequences. Node values denote the percentage of 100 bootstrap replicates recovered for that particular bipartition. (PDF) [file pone.0033261.s004.pdf]

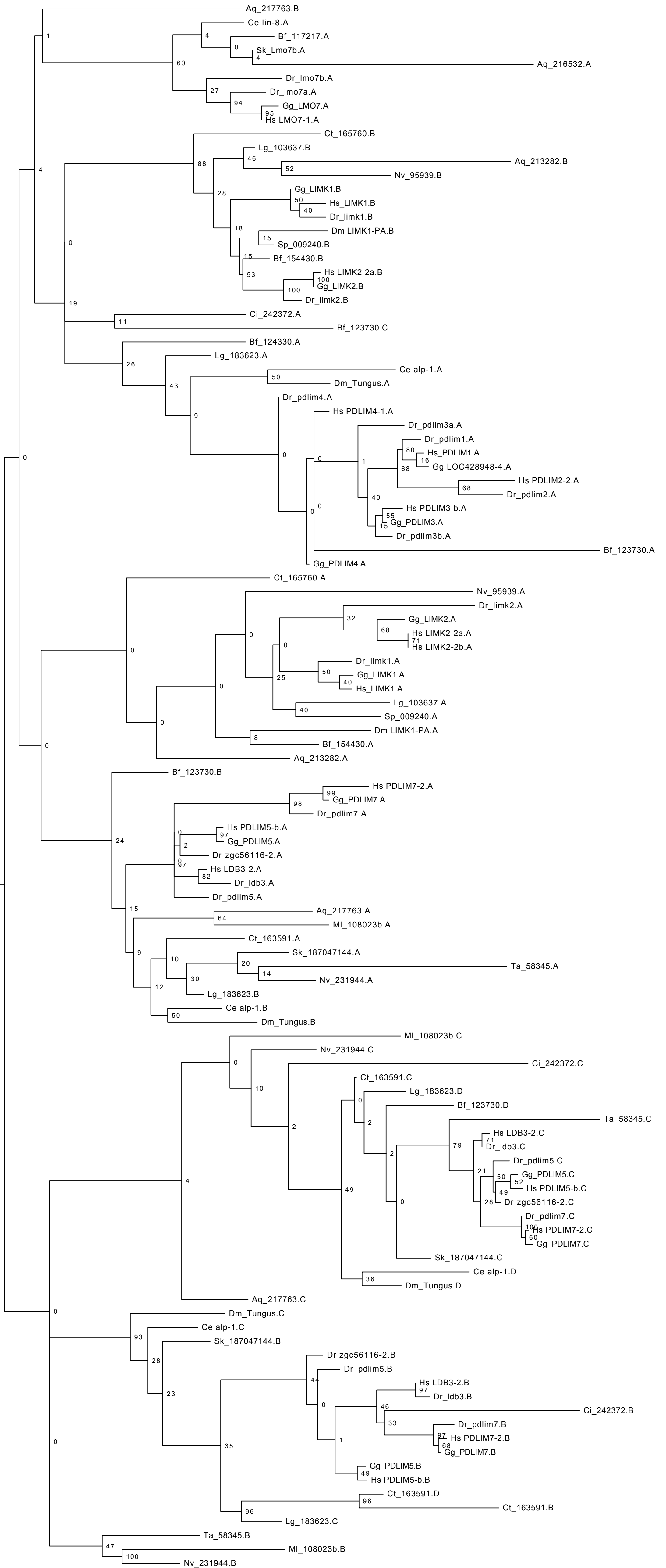

0.3

Supplement: Figure S7 — LIM domain tree from ENIGMA, LIMK, and LMO7 class proteins. Midpoint rooted phylogram of ENIGMA, LIMK, and LMO7 class LIM domain phylogeny (maximum likelihood). See Table S1 for details on individual sequences. Node values denote the percentage of 100 bootstrap replicates recovered for that particular bipartition. (PDF) [file pone.0033261.s007.pdf]
