# Supplementary material for: The Diversification of the LIM Superclass at the Base of the Metazoa Increased Subcellular Complexity and Promoted Multicellular Specialization
Source: PLoS One. 2012 Mar 15;7(3):e33261. doi: 10.1371/journal.pone.0033261 (PMC3305314; doi:10.1371/journal.pone.0033261)
Supplement: Figure S2 — LIM domain tree. Midpoint rooted phylogram of LIM domain phylogeny (maximum likelihood). Alternating blue and grey coloring delineates homology groups; black regions are unclassified. Conserved LIM group labels appear within the upper edge of a clade. See Figure 2 for more details on homology groups and tree labeling. See Table S1 for details on individual sequences. See Table S1 for the corresponding alignment. Node values denote the percentage of 100 bootstrap replicates recovered for that particular bipartition. (PDF) [file pone.0033261.s002.pdf]

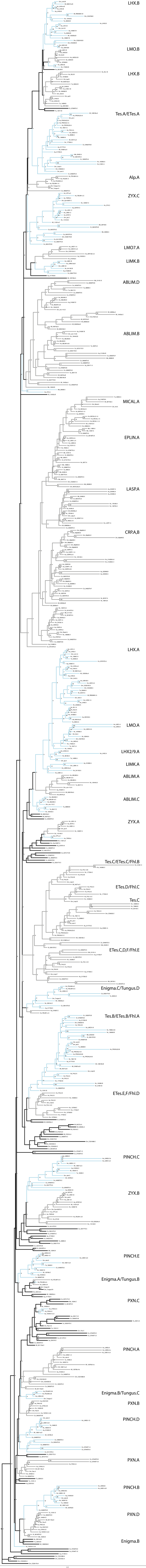
LHX.B
LMO.B
LHX.B
Tes.A/ETes.A
Alp.A
ZYX.C
LMO7.A
LIMK.B
ABLIM.D
ABLIM.B
MICAL.A
EPLIN.A
LASP.A
CRP.A,B
LHX.A
LMO.A
LHX2/9.A
LIMK.A
ABLIM.A
ABLIM.C
ZYX.A
Tes.C/ETes.C/Fhl.B
ETes.D/Fhl.C
Tes.C
ETes.C,D,F/Fhl.E
Enigma.C/Tungus.D
Tes.B/ETes.B/Fhl.A
ETes.E,F/Fhl.D
PINCH.C
ZYX.B
PINCH.E
Enigma.A/Tungus.B
PXN.C
PINCH.A
Enigma.B/Tungus.C
PXN.B
PINCH.D
PXN.A
PINCH.B
PXN.D
Enigma.B
